# Theca Cell INSL3 and Steroids Together Orchestrate the Growing Bovine Antral Follicle

**DOI:** 10.3389/fphys.2017.01033

**Published:** 2017-12-12

**Authors:** Yanzhenzi Dai, Richard Ivell, Ravinder Anand-Ivell

**Affiliations:** ^1^School of Biosciences, University of Nottingham, Nottingham, United Kingdom; ^2^School of Veterinary Medicine and Science, University of Nottingham, Nottingham, United Kingdom; ^3^School of Biological Sciences, University of Adelaide, Adelaide, SA, Australia

**Keywords:** insulin-like peptide 3, RXFP2, testosterone, androstenedione, estradiol, ovarian theca cells, PCOS

## Abstract

Insulin-like peptide 3 (INSL3) and its specific receptor RXFP2 are both expressed by theca interna cells of the growing antral follicle where they form an essential regulatory element in the production of the steroid precursor androstenedione. Using primary cultures of bovine theca cells from the mid follicular phase together with steroid agonists and antagonists we have examined how ovarian steroids modulate INSL3 expression. Transcript analysis shows that these cells express estrogen receptors α and β, androgen and progesterone receptors, besides the orphan nuclear receptors SF1 and nur77. Whereas, exogenous androgens have little or no effect, the androgen antagonist bicalutamide stimulates INSL3 production. In contrast, estrogen receptor agonists, as also progesterone, are stimulatory. Importantly, estrogen receptor signaling is convergent with the protein kinase A signaling pathway activated by LH, such that the estrogen receptor antagonist can inhibit the mild stimulatory effect of LH, and vice versa the PKA antagonist H89 blocks stimulation by estradiol. A significant finding is that the major steroid metabolite androstenedione appears to act predominantly as an estrogen and not an androgen in this system. Transfection of INSL3 gene promoter-reporter constructs together with various steroid receptor expression plasmids supports these findings and shows that steroid action uses non-classical pathways not requiring canonical steroid-responsive elements in the proximal promoter region. Together, the results indicate that increasing estrogens in the follicular phase stimulate a feedforward loop driving INSL3 signaling and thereby promoting steroidogenesis in the growing antral follicle until the LH surge which effectively switches off INSL3 expression.

## Introduction

According to the “two-cell two-gonadotropin” hypothesis, the two major somatic cell types of the ovarian antral follicle mutually interact to attain coordinated and optimized function during the estrous cycle, leading to the ovulation of a mature and fertile oocyte (Hillier et al., 1994). Part of this interaction involves the production of steroid hormones by both cell types, respectively the theca interna cells (TC) and the granulosa cells (GC). In the small (3–5 mm diameter) to medium (6–8 mm diameter) follicles of the mid cycle, prior to the LH surge, TC produce principally androstenedione (A4) via the Δ5 pathway, but also smaller amounts of testosterone and DHEA, as well as some progesterone via the Δ4 pathway (Berndtson et al., 1995; Spicer, 2005). Whilst TC do use cholesterol to produce the major precursor pregnenolone, it is believed that much of TC steroidogenesis derives from pregnenolone exported from GC (Fortune, 1986), which reciprocally import androstenedione and testosterone from TC, and using the enzyme aromatase convert these into estradiol and estrone. Later, following the LH surge, both GC and TC dedicate metabolism to the production via the Δ4 pathway of progesterone in the process known as luteinisation. The “two-cell two-gonadotropin” hypothesis states that while the mid-cycle TC are governed uniquely by the pituitary gonadotropin LH, mid-cycle GC rely more or less exclusively on FSH, although some LH receptors are beginning to be expressed there. Thus, the growing antral follicle of the cycle is a dynamic steroidogenic tissue whose physiology is dominated by the reciprocal interactions of GC and TC. These features are common to both human and bovine antral follicles, and the bovine system is recommended as an excellent model for the equivalent physiological events in the human (Adams et al., 2012).

Another important facet of the growing antral follicle is that while the TC layer is supplied by vigorous capillary vascularization, the GC layer is effectively segregated from the circulation by a dense basal layer. Moreover, follicular fluid accumulates high levels of steroids from both GC and TC, which govern the environment of the oocyte as well as the follicular somatic cells. Steroid concentrations may be very high, in the region of 10^−7^ to 10^−6^ molar or even greater (Ivell et al., 2014). This is a concentration which is well above the dynamic range for conventional nuclear steroid receptors (10^−10^ to 10^−8^ molar) but nevertheless appears to regulate important ovarian functions such as follicle transition and maturation (Kezele and Skinner, 2003; Yang and Fortune, 2006). It seems likely that such follicular steroids are making use of non-classical receptor mechanisms, which may operate in a dynamic range with 10 to 100-fold less sensitivity than for classical steroid receptor systems (Ivell et al., 2014).

The growing ovarian follicle additionally produces a number of mostly autocrine/paracrine acting peptide hormones and other compounds. These include from GC, oxytocin, AMH, relaxin, inhibin, activin, components of the renin-angiotensin and natriuretic peptide systems, as well as BMPs (Bagnell, 1991; Stormshak et al., 1995; Yoshimura, 1997; Webb et al., 2003; Knight et al., 2012), and from TC, the insulin-like peptide INSL3 (Bathgate et al., 1999; Dai et al., 2017). All of these hormone-receptor systems aid in orchestrating the growth and selection of an antral follicle, and the maturation of an enclosed oocyte, leading to ovulation and the synchronous preparation of the oviduct and uterus for pregnancy.

As a model system we are using here primary cultures of TC isolated from medium-sized (6–8 mm diameter) healthy antral follicles of bovine ovaries lacking a corpus luteum and collected from a local slaughterhouse. As such we can be confident that these TC will not yet have seen *in vivo* the high LH concentration of the LH surge and hence not have begun luteinisation. Moreover, we have shown previously that such TC express the gene for INSL3 and secrete INSL3 peptide into the culture medium (Bathgate et al., 1999; Glister et al., 2013). INSL3 is part of an essential autocrine-paracrine network which by activating its specific receptor (called RXFP2) on other TC (Dai et al., 2017) is able to regulate and orchestrate the production of androstenedione (Glister et al., 2013). Since this is the main steroid precursor for GC, this means that INSL3 is responsible for most steroidogenesis within the growing antral follicle. Moreover, we have previously shown that the profile of INSL3 secretion by waves of growing antral follicles is similar between humans and cows (Anand-Ivell et al., 2013; Satchell et al., 2013) suggesting that these properties are likely shared between the two species. The objective of the present study is to investigate whether and by how much INSL3 expression by bovine TC can itself be modulated by follicular steroids and the molecular mechanisms that may be involved in this. It has been shown for the mouse Leydig cell-line MA-10, for example, that the INSL3 gene can be modulated by estradiol (Lague and Tremblay, 2009) and by testosterone (Lague and Tremblay, 2008), even though there are no conventional steroid-responsive elements within the proximal promoter region of the mouse INSL3 gene (Koskimies et al., 1997). Rather, it appears that the estrogen action may involve the essential binding of the orphan receptor steroidogenic factor-1 (SF1), as has been shown previously for the basal expression of this gene in mice, rats, and human (Koskimies et al., 1997; Sadeghian et al., 2005; Tremblay et al., 2009).

Together the results presented here for theca cells of growing antral follicles show that INSL3 is part of an essential ovarian feedforward loop driving steroidogenesis and increasing estrogen production during the follicular phase until halted by the LH surge.

## Materials and methods

### Cell preparation, culture, and treatment

The isolation of bovine TC essentially followed Glister et al. (2013) except that cells were cultured on uncoated plates and flasks. Briefly, bovine ovaries were collected from the local slaughterhouse in accordance with German, British and European ethical regulation. They were transported to the laboratory in warm DPBS (Dulbecco's phosphate-buffered saline; Gibco/Thermo-Fisher, Waltham, MA) with 2% antibiotic-antimycotic (ABAM; Sigma-Aldrich, Dorset, UK). Follicles of 4–6 mm diameter were dissected from ovaries without an obvious corpus luteum and granulosa cells removed. The TC layer was peeled away and digested in Medium 199 (Sigma) with additional collagenase IV (1 mg/ml; Sigma) and trypsin inhibitor (100 μg/ml; Sigma) in a shaking water bath at 38°C. After 45 min, cells were dissociated by repeated pipetting and digestion continued for a further 15 min. After 1 × g sedimentation for 2 min the liquid phase was transferred to Medium 199 with 1% ABAM. After brief centrifugation, cells were re-suspended in DPBS and any red blood cells removed by rapid hypotonic shock; the resulting cells were re-suspended in TC culture-medium [McCoy's 5A medium (Sigma), with 1% ABAM, 2 mM L-glutamine (Gibco), 10 mM HEPES (Sigma), 0.1% BSA (Biomol GmbH, Hamburg, Germany), 10 ng/ml bovine insulin (Sigma), 5 μg/ml bovine apo-transferrin (Sigma), and 5 ng/ml sodium selenite (Sigma)] and maintained at 38°C with 5% CO_2_. TC were seeded at ~180,000 cells/well (48-well plate) and incubated for 30 h, then changed to fresh TC culture-medium containing stimulants, followed by incubation for a further 48 h at 38°C with 5% CO_2_. The inhibitors [onapristone and RU 486 (kind gifts from Professor Walter Elgar, Berlin, Germany), bicalutamide (Sigma), ICI-182,780 (ICI; Tocris Bioscience, Bristol, UK), and H89 (Sigma)] were then added, followed by estradiol (E2; Sigma), androstenedione (A4; Fluka, Steinheim, Germany), 5 alpha-dihydrotestosterone (DHT; Sigma), 8Br-cAMP (Sigma), or LH from sheep pituitary (Sigma), as indicated in the figures. After a further 48 h, the culture-medium was collected and stored at −20°C until INSL3 measurement (see below). All treatments were performed in triplicate. Finally, the cells from triplicate wells were washed with DPBS and pooled in TriZol (Ambion/Thermo-Fischer, Waltham, MA) for RNA extraction (see below). All experiments were repeated independently at least three times. To test for toxicity, TC were seeded at ~80,000 cells/well (96-well plate), and cultured for 30 h at 38°C with 5% CO_2_. Chemicals were then applied for 48 h and cell viability measured using the MTT (3-(4, 5-dimethylthiazolyl-2)-2,5-diphenyltetrazolium bromide) assay (Invitrogen/Thermo-Fischer, Waltham, MA), following the manufacturer's protocol.

### Bovine INSL3 immunoassay

Bovine INSL3 secreted into the culture medium was measured by a specific time-resolved fluorescent immunoassay (TRFIA) previously described in detail and validated by us (Anand-Ivell et al., 2011; Glister et al., 2013).

### mRNA isolation and RT-PCR

RNA was separated from the TriZol extracts by addition of 0.2 vol chloroform (Sigma) and centrifugation at 2,000 × g for 10 min; the aqueous phase containing the RNA was precipitated with 0.5 vol isopropanol (Sigma), pelleted by centrifugation and washed twice in 80% EtOH. The RNA pellet was air-dried, dissolved in RNAse-free water (Ambion) and stored in −80°C. To avoid any DNA contamination, the extracted RNA was treated using the TURBO DNAse-free kit (Ambion), adhering to the manufacturer's protocol. RNA quantity and quality was assessed by UV spectrophotometry at 260 and 280 nm and by gel electrophoresis to visualize the integrity and ratio of the 18S and 28S ribosomal RNA.

cDNA was prepared using the Superscript III reverse transcriptase kit (Invitrogen /Thermo-Fischer, Waltham, MA) primed with oligo (dT)_12−18_ (Invitrogen) following the manufacturer's protocol. The synthesized cDNA was diluted 1:5 with RNAse-free water and stored at −20°C. RT-PCR was carried out using the primers listed in Table 1. DNA was denatured at 95°C for 45 s, annealing was for 1 min at the temperature indicated in Table 1, with elongation for 1 min at 72°C, for a total of 35 cycles. As positive control, all samples were checked for the expression of the housekeeping gene bovine S27a (Anand-Ivell et al., 2006). 0.25 μM of the primer pair (Eurofins Genomics, Ebersberg, Germany) were used to amplify 1 μl of cDNA, using the Takara Sybrgreen Premix II (Takara, Shiga, Japan) for real-time qRT-PCR in a Rotor-Gene 3000 machine (Corbett Research, Mortlake, Australia). PCR products were validated by melt curves and by gel electrophoresis of the final product. The real-time qRT-PCR results were normalized against the bovine S27a transcript (Anand-Ivell et al., 2006).

**Table 1:** List of oligonucleotide primers used for RT-PCR.

**Transcript**	**No**.	**Orientation**	**Sequence (5′-3′)**	**Annealing Temp (°C)**	**Accession No**.
S27a	501	Forward	tcctcctgaccagcaaagactg	62	NM_174778
	502	Reverse	tgcttgttcttcttgggagtgg		
ERα	566	Forward	agggaagctcctatttgctcc	62	NM_001001443
	567	Reverse	cggtggatgtggtccttctct		
ERβ	568	Forward	gcttcgtggagctcagcctg	62	NM_174051
	569	Reverse	aggatcatggccttgacacaga		
AR	570	Forward	cagatggcagtcattcag	60–54	NM_001244127
	571	Reverse	cttggtgagctggtagaag	touchdown	
PR	593	Forward	gggatgaagcatcaggctgt	61	NM_001205356
	594	Reverse	ttctctggcttagggcttgg		
SF1	599 600	Forward Reverse	agctgctggtttttgaccac cgaggctgaagagaatgagg	60	NM_174403
nur77	597 598	Forward Reverse	ctgtgtgtggggacaatgct gcatcctctttcccgaagtg	61	NM_001075911
INSL3	574	Forward	gcgcctgagaaactgtgtgg	62	NM_174365
	575	Reverse	gggttgatggcagttgctcg		

*Oligonucleotide primers were designed using the Primer3 software and the transcript sequences indicated by the accession numbers*.

### Bovine INSL3 gene promoter-reporter constructs and theca cell transfection

The sequence of the bovine INSL3 gene and upstream promoter region was selected from the latest release of the bovine genome project for chromosome 7 (acc. no. AC_000164.1). In analogy to the characterized promoter regions for the human and rat sequences (Sadeghian et al., 2005; Tremblay et al., 2009) the proximal promoter region of the bovine INSL3 gene was considered to be limited in the 5′ direction by the location of the last exon 23 of the JAK3 gene. The 1,800 bp upstream promoter sequence of the bovine INSL3 gene was independently verified by PCR and sequencing from bovine genomic DNA (unpublished and Suhrbier, 2000). This sequence, terminating 3′ at the putative transcription start site, was inserted into the multiple cloning site of the reporter construct pGL3-B (Promega, Southampton, UK) to create the full-length promoter-reporter construct bI3_1L. A second full-length construct (bI3_FL) was also created which additionally included a further 10 nucleotides of the 5′ UTR of the INSL3 mRNA sequence. Subsequently, a series of seven 5′ deletion promoter fragments of the bI3_1L construct were prepared (bI3_2L to bI3_8L). All constructs were custom-made and verified by GeneArt (GeneArt/Thermo-Fischer, Waltham, MA). Expression constructs for the transcription factors human estrogen receptor alpha (ERα), and beta (hERβ) bovine ERβ (bERβ), bovine SF1 (all a generous gift from Dr Norbert Walther, Hamburg, Germany, and in the vector pRcCMV) and human androgen receptor (AR) (a kind gift from Dr Yvonne Giwercman, Lund, Sweden, also in a CMV-driven promoter construct, AR-pCMV4) were as previously described (Stedronsky et al., 2002; Nenonen et al., 2010).

For transfection, the TC were cultured at a density of 1.6 million cells per T75 flask for 18 h. The cells were then trypsinised and washed twice with DPBS (without Ca^2+^ or Mg^2+^) (Gibco) and transfected using the Neon transfection system (Invitrogen), following the manufacturer's instructions. Transfection conditions for TC were first optimized using a GFP-expression plasmid followed by microscopic analysis (not shown). Cells were transfected with varying combinations of DNA at a ratio of 6 μg DNA/500,000 cells. Between 0.5 and 2 million cells were transfected in 100 μl of Buffer R in a 100 μl Neon tip using a single pulse of 1,500 volts for 20 milliseconds. After each transfection, the cells were diluted in antibiotic-free medium at 250,000 cells/500 μl/well (48-well plate). Unless otherwise stated, the ratio of promoter-reporter (luciferase) plasmid: receptor expression plasmid: LacZ expression vector was 2: 3: 1. As negative control for the promoter-reporter plasmids, the pGL3-B plasmid was used which contains neither promoter nor transcriptional enhancer sequences. The empty vector p0GH was used to make up any DNA deficit. Transfected cells were then treated with various stimulants and cultured for 24 h in antibiotic-free culture-medium. After removal of the medium, the cells were lysed in 70 μl of 1×passive lysis buffer (Promega) and the transcriptional activity of promoters determined by measuring luciferase activity in 25 μl of cell lysate using the Luciferase Assay System (Promega) following the manufacturer's instructions. Luminescence was measured immediately using a SIRIUS Luminometer (Berthold, Pforzheim, Germany) and expressed as Relative Light Units (RLU) in relation to β-galactosidase expression of co-transfected LacZ expressing plasmids measured in 25 μl of cell lysate using the β-galactosidase Enzyme Assay System (Promega). For each experiment, at least three replicates were used per treatment, and each set of treatments were repeated independently 2–4 times.

### Statistics and analysis

All data were analyzed using the Graphpad Prism version 6 package. For primary TC experiments results were analyzed using one-way ANOVA followed by Dunnett's multiple comparisons test.

## Results

### Basal responsiveness of cultured bovine TC to LH or 8Br-Camp

**Figure 1:**
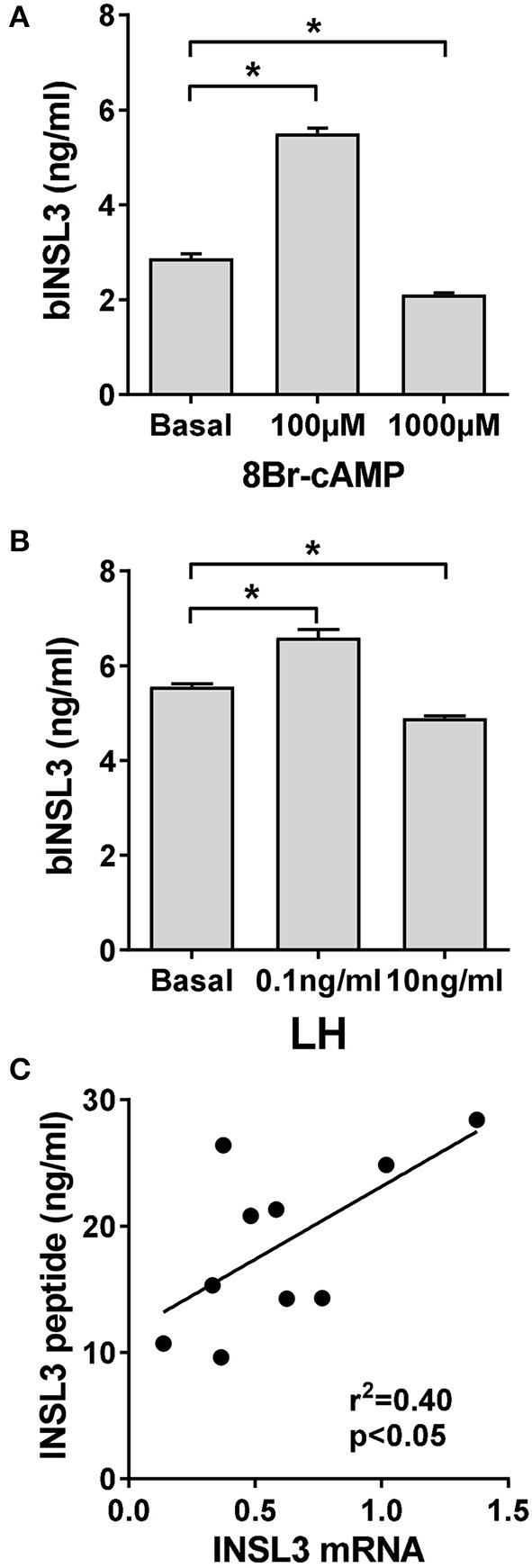
Effect of 8Br-cAMP or LH on INSL3 production by bovine theca internal cells. **(A)** Effect of low and high-dose 8Br-cAMP. **(B)** Effect of low and high-dose LH. **(C)** Correlation between secreted INSL3 peptide concentration and INSL3 mRNA measured by qRT-PCR in the same cells within the same experiment. ^*^Indicates significant difference *p* < 0.05.

All TC cultures are treated or not with steroids or their inhibitors for 48 h before measuring the cell response. In this time, TC will also be generating limited steroids such as androstenedione, progesterone, DHEA or testosterone (Berndtson et al., 1995; Spicer, 2005), hence in subsequent experiments steroid receptor antagonists are used to determine effects of such endogenous steroids. Furthermore, it is important within this time-frame to recognize the difference between effects of low-dose LH (or 8Br-cAMP) equivalent to mid-cycle *in vivo* concentrations and high-dose LH (or 8Br-cAMP) equivalent to the levels experienced during the LH surge and which lead to luteinisation (Berndtson et al., 1995). Low-dose LH (100 pg/ml), like low-dose 8Br-cAMP (100 μM) leads to only a modest increase in secretion of bINSL3 into the culture medium (Figures 1A,B). In contrast, high dose 8Br-cAMP (1 mM), like high-dose LH (10 ng/ml), leads to an inhibition of bINSL3 secretion (Figures 1A,B). It should be noted that we are using the accumulation of the peptide bINSL3 into the culture medium as a surrogate for bINSL3 gene transcription. That this is justified can be seen in Figure 1C, where a good correlation (*p* < 0.05) is seen between bINSL3 in the culture medium and levels of bINSL3 mRNA measured by qRT-PCR within one experiment.

### Expression of steroid receptors in cultured bovine TC

**Figure 2:**
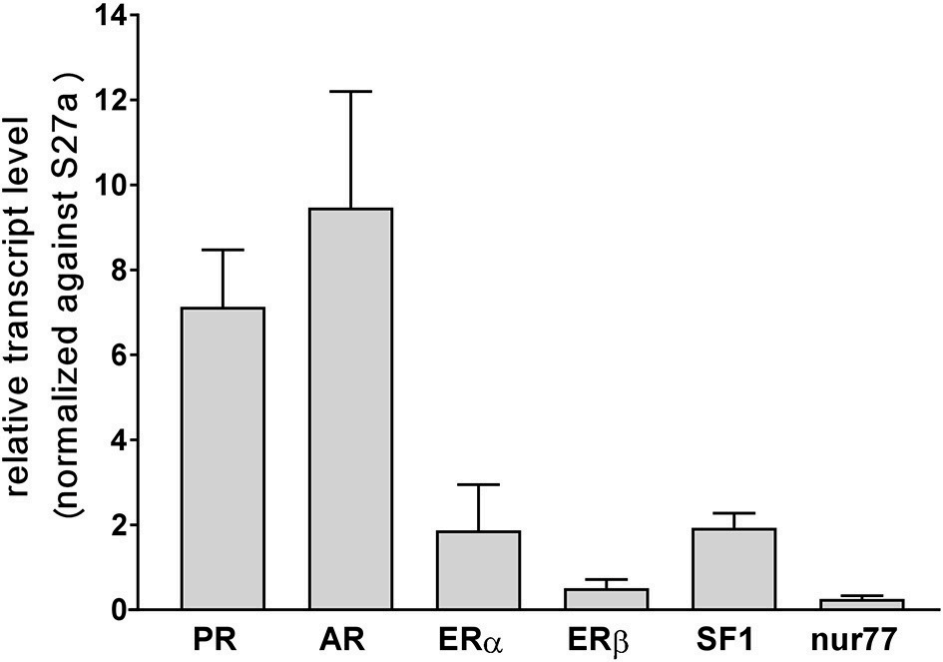
Relative transcript levels (×10^−2^) for the steroid receptors PR, AR, ERα, and ERβ, as well as for the orphan nuclear receptors SF1 and nur77, measured by qRT-PCR and normalized against bovine S27a transcripts, for three independent batches of primary cultures of bovine theca interna cells under basal conditions.

qRT-PCR was used to verify the relative expression of transcripts for the nuclear steroid receptors for progesterone (PR), estrogens (ERα and ERβ), and androgens (AR) in the bovine TC under basal culture conditions (Figure 2). All four receptors appear to be expressed in these cells, with highest levels seen for AR, and lowest for ERβ. Similarly, the expression levels of transcripts for the two orphan nuclear receptors, considered most relevant for steroidogenic enzyme expression, SF1 (NR5A1) and nur77 (NR4A1) were also measured (Figure 2), whereby both were not as highly expressed as PR or AR, and nur77 was at a very low level in these mid-follicular phase theca cells.

### Impact of estradiol and estrogen receptor antagonist on INSL3 expression in bovine TC

**Figure 3:**
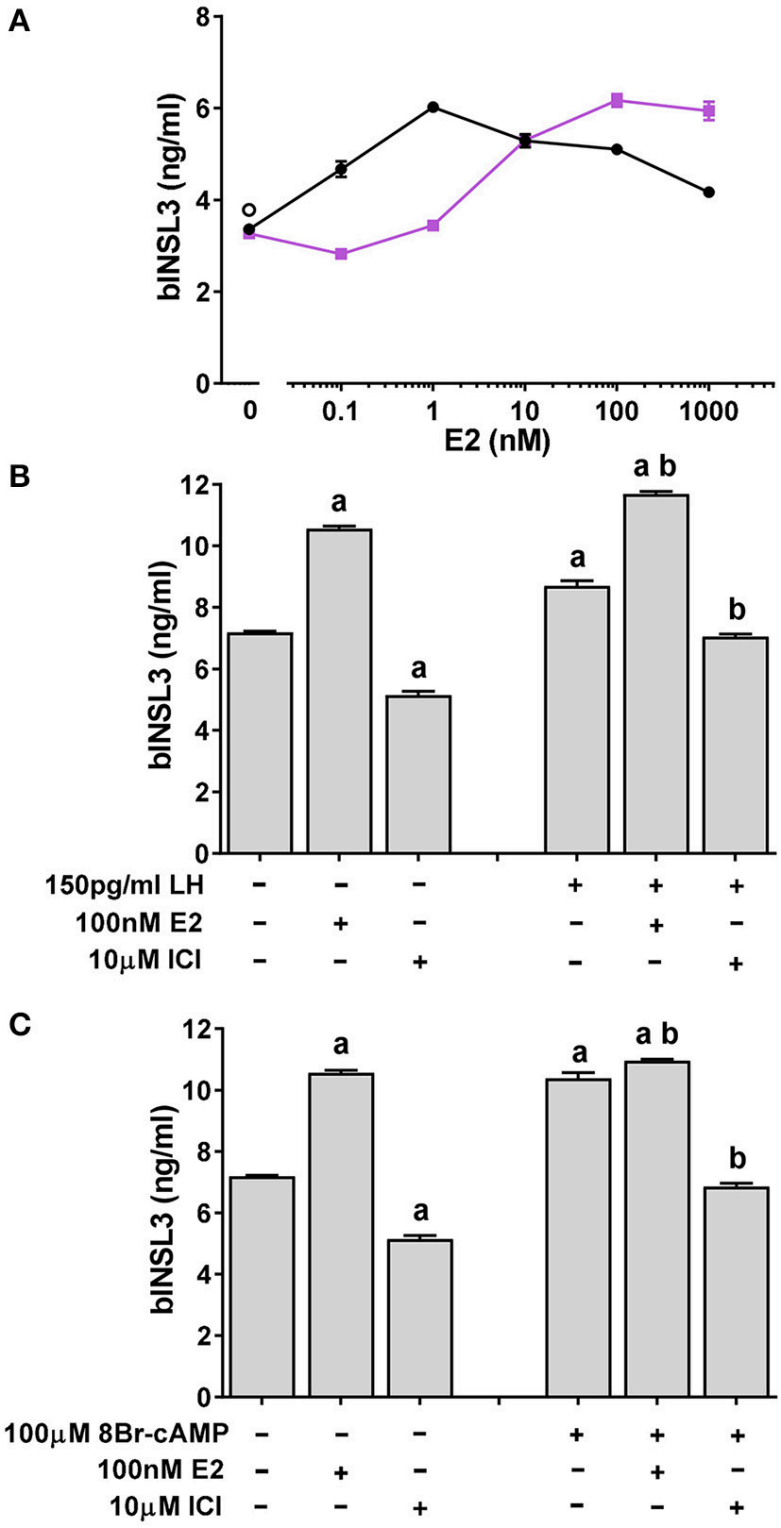
Effect of estrogen receptor agonist (estradiol; E2) and antagonist (ICI-182,780; ICI) on basal and low-dose LH or 8Br-cAMP stimulated INSL3 production by bovine theca internal cells. **(A)** E2 dose-response in the absence (black circles) or presence (pink squares) of 10 μM ICI. **(B)** Effect of E2 and/or ICI on low-dose LH stimulated INSL3 production. **(C)** Effect of E2 and/or ICI on low-dose 8Br-cAMP stimulated INSL3 production. Statistical significance *p* < 0.05: a, vs. basal control; b, vs. LH or 8Br-cAMP control.

Because aromatase is not expressed in TC under basal conditions, culture medium should be free of estradiol, estrone or other estrogens. However, *in vivo*, TC will be exposed to estrogens coming from the GC compartment. Addition of estradiol to TC shows a bell-shaped dose-response curve (Figure 3A) with a maximum at 1 to 100 nM. Addition of the pure estrogen receptor antagonist ICI-182,780 leads to complete inhibition of such stimulation at low doses of estradiol, though stimulates at high doses, the dose-response curve being shifted markedly to the right.

However, when ICI-182,780 alone is added to basal TC, there is an inhibition of bINSL3 production (Figure 3B). Further addition of low-dose LH does little to relieve this inhibition (Figure 3B), implying that even in the absence of exogenous estrogens, their specific nuclear receptor(s) appear to be obligatorily involved in bINSL3 expression. The same is true also when LH is replaced by low-dose 8Br-cAMP (Figure 3C). Note that estradiol has little if any additive effect to that of 8Br-cAMP, in contrast to its effect on LH.

**Figure 4:**
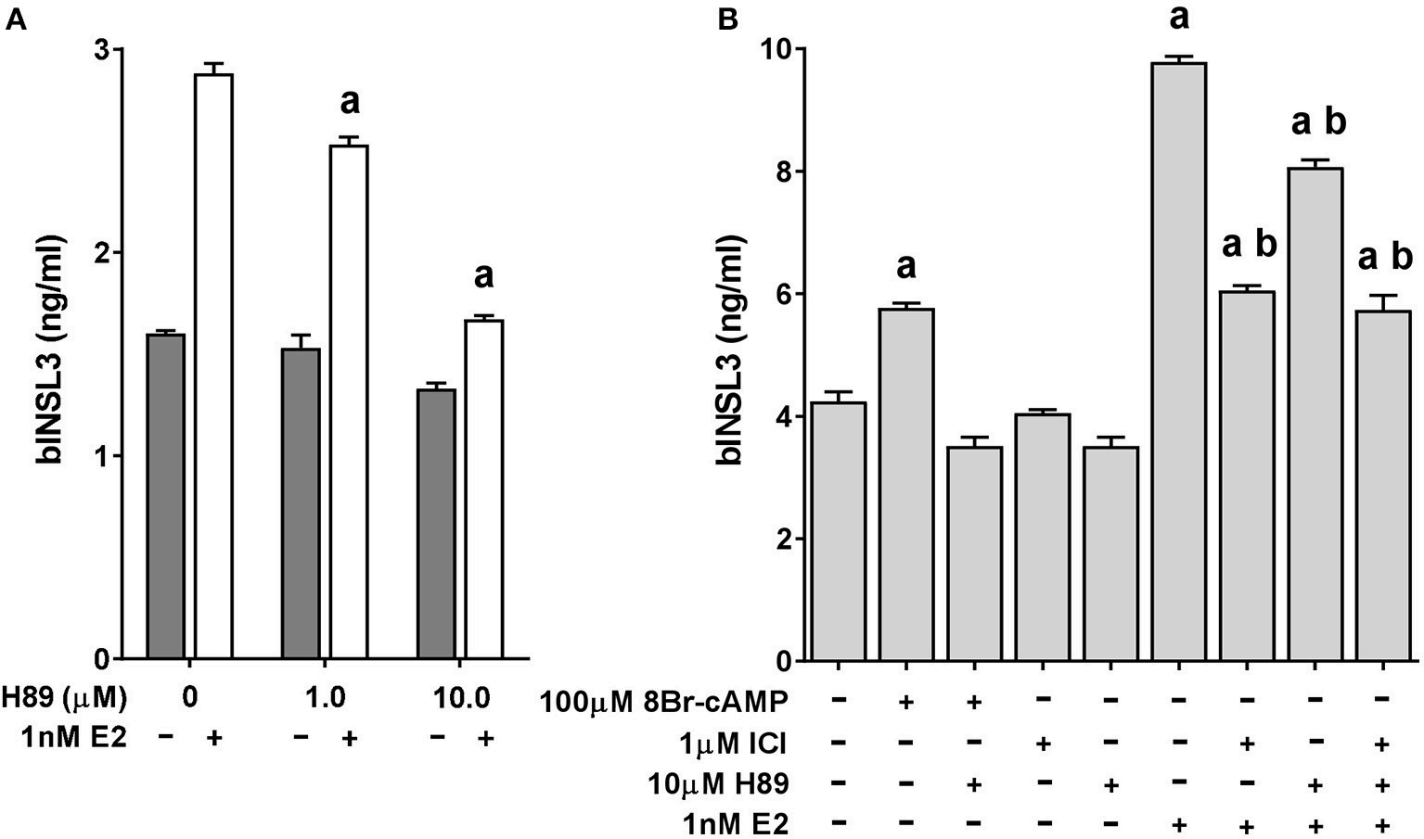
Effect of estrogen receptor agonist (estradiol; E2) and antagonist (ICI-182,780; ICI) on INSL3 production by bovine theca interna cells in the presence or absence of the PKA inhibitor H89. **(A)** Dose-response for H89 in the presence (white bars) or absence (dark bars) of 1 nM E2. **(B)** Effect on INSL3 production of 10 μM H89 on basal, 8Br-cAMP, ICI and estradiol treated bovine theca interna cells. Statistical significance *p* < 0.05: a, vs. basal control; b, vs. E2 stimulated control.

To determine at what level the pathways involving LH or 8Br-cAMP and the estrogen receptor might interact, TC cultures were additionally treated with the protein kinase A (PKA) inhibitor H89 (Figure 4). Both 1 and 10 μM H89 led to a significant inhibition of the estradiol-induced stimulation (Figure 4A), without affecting cell viability, although 50 μM H89 proved toxic to the cells (not shown). However, there was no further inhibition beyond that imposed by addition of 1 μM ICI-182,780 (Figure 4B). As a control, H89 was shown to completely block the stimulatory effect of low-dose 8Br-cAMP (Figure 4B).

### Impact of androgens on INSL3 expression in bovine TC

**Figure 5:**
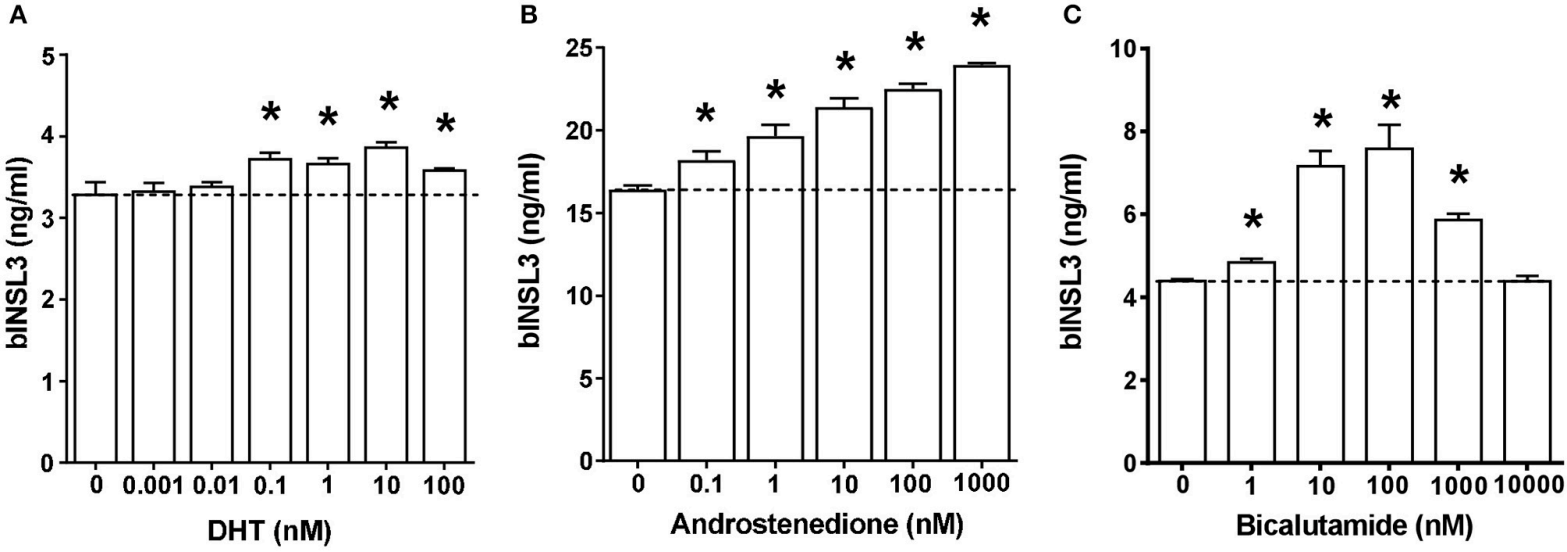
Dose-response effects of androgen receptor agonists (**A**, dihydrotestosterone, DHT. **B**, androstenedione) and antagonist (**C**, bicalutamide) on INSL3 production by bovine theca interna cells. ^*^Significantly different from basal control, *p* < 0.05.

Addition of the relatively non-metabolisable androgen dihydrotestosterone (DHT) indicated only a modest stimulatory effect (Figure 5A). Only 4 of 5 independent experiments showed a significant increase above basal of 1.26 ± 0.23 (mean±SD) fold at 1 nM DHT. Also androstenedione (Figure 5B) showed a small but significant stimulation on basal bINSL3 production (5 of 6 independent experiments; 1.22 ± 0.16 (mean±SD) fold over basal at 10 nM androstenedione). However, when TC were treated with the specific anti-androgen bicalutamide (Figure 5C), production of bINSL3 was markedly stimulated with a bell-shaped response, though again only in 4 from 6 independent experiments each at 10 or 100nM. Remaining experiments showed no effect. This androgen receptor antagonist is presumably inhibiting the activity of any endogenous androgens. The positive effect of androstenedione at <10 nM was not significantly influenced by 100 nM bicalutamide, though could be blocked by 1 μM ICI-182,780 (not shown).

### Impact of glucocorticoids and gestagens on INSL3 expression in bovine TC

**Figure 6:**
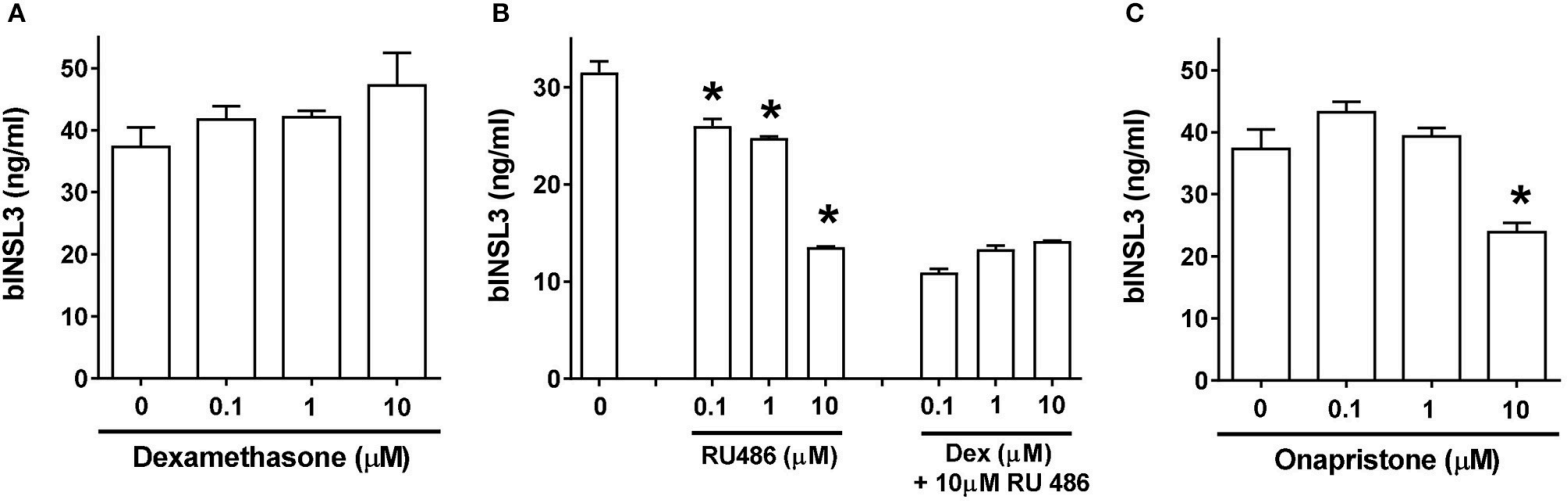
Dose-response effects of the glucorticoid/progesterone receptor agonists (**A**, dexamethasone, Dex) and antagonists (**B**, RU486 and **C**, onapristone) on INSL3 production by bovine theca interna cells. ^*^significantly different from basal control, *p* < 0.05.

TC are known to generate a certain amount of endogenous progesterone (Berndtson et al., 1995), besides being exposed to gestagens and glucocorticoids *in vivo*. In order to check for effects of these steroids on bINSL3 production, cultured TC under basal conditions were treated with the progesterone/glucocorticoid receptor antagonist RU486 (Figure 6B). There is a marked dose-dependent inhibition implying the involvement of either activated PR or GR in bINSL3 production. However, the glucocorticoid dexamethasone is completely without effect at various concentrations (Figure 6A), nor does it reverse the inhibitory effect of RU486 (Figure 6B). A similar inhibition was also seen when using the pure PR antagonist onapristone (Figure 6C), confirming that activated PR rather than GR is involved in the expression of INSL3 in bovine theca cells.

### Analysis of the proximal promoter of the bovine INSL3 gene

**Figure 7:**
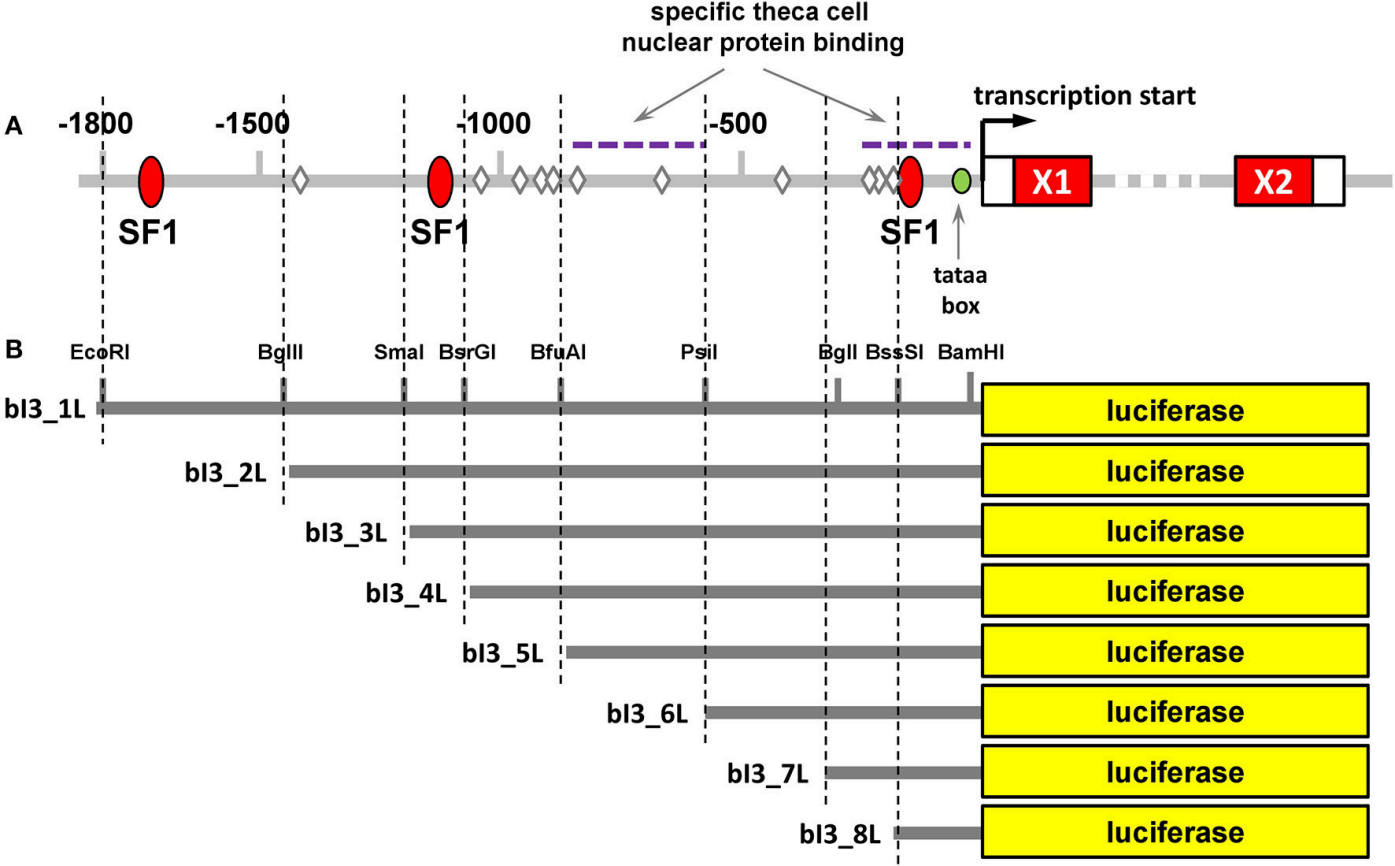
**(A)** Bioinformatic analysis of the upstream promoter region of the bovine INSL3 gene analyzed using TRANSFAC software. Red ellipses indicate putative binding sites for the transcription factor steroidogenic factor 1 (SF1); diamonds indicate putative steroid receptor half-sites. Exon 1 (X1) and exon 2 (X2) are indicated as red oblongs on the right. **(B)** Relative positions of the deletion promoter-reporter constructs used for the transfection experiments including relevant restriction enzyme cleavage sites.

Approximately 1,800 bp of the proximal promoter region sequence upstream of the first exon of the bovine INSL3 gene on chromosome 7 and immediately downstream of the JAK3 gene was derived from the latest edition of the bovine genome project. This sequence was confirmed also by genomic cloning and sequence analysis (unpublished and Suhrbier, 2000). Bioinformatic analysis of this promoter region identified three potential sites for SF-1 binding (Figure 7) though no other consensus motifs representing canonical steroid receptor responsive elements. There were, however, several elements representing so-called half-sites. Promoter-reporter constructs were made in the vector pGL3-B. These included a full-length promoter and a series of deletion constructs as indicated (Figure 7B). Two full-length constructs had been made which differed only in their 3′ sequences: bI3-1L, like all deletion constructs ended at the transcription start site, whereas bI3-FL additionally included 10 nucleotides of the 5′ UTR of the first exon. In all subsequent analyses bI3-1L and bI3-FL behaved identically.

**Figure 8:**
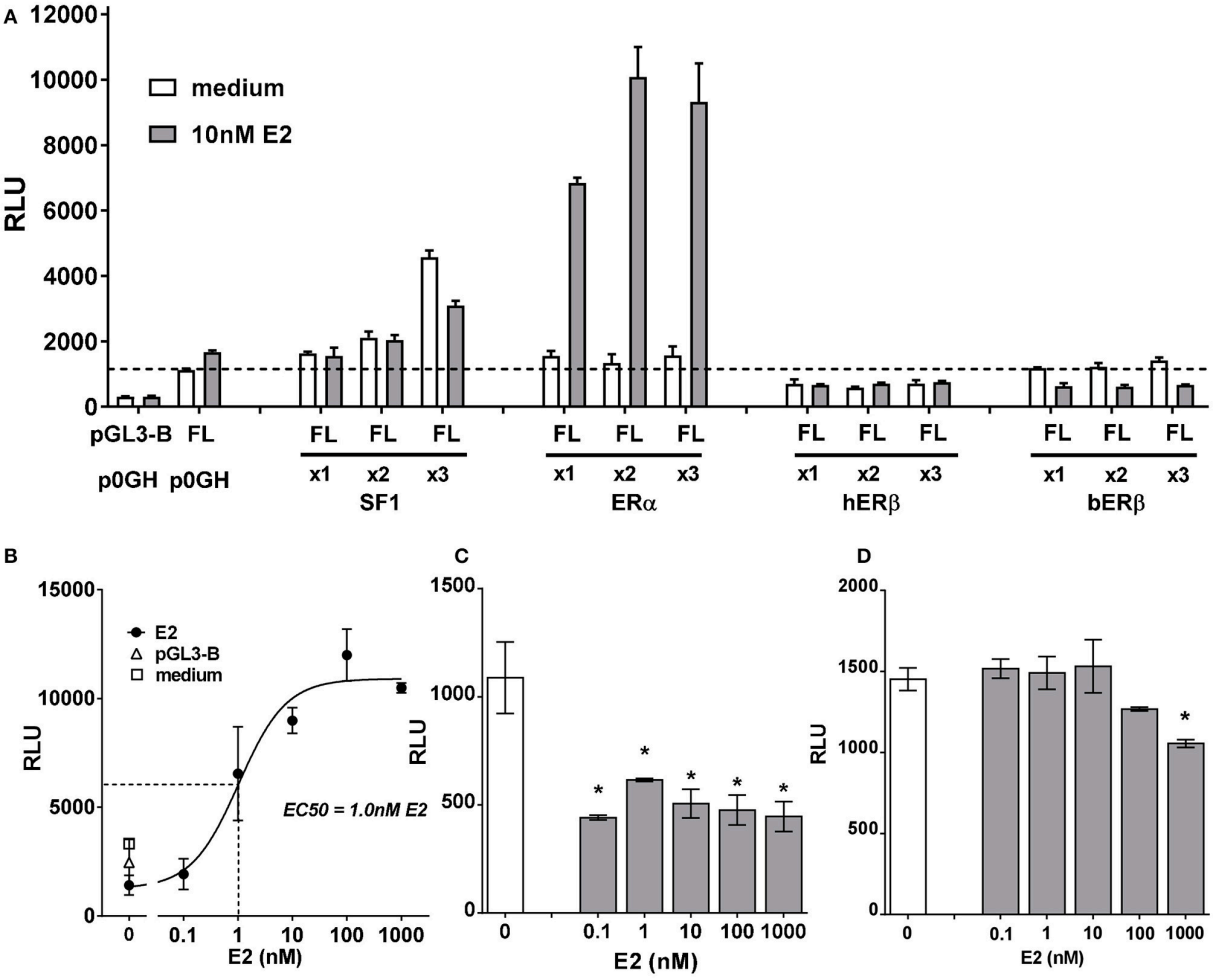
**(A)** Luciferase activity (relative light units, RLU) generated in bovine theca interna cells transfected with the full-length INSL3 promoter-reporter construct (bI3-FL) in the presence or absence, as indicated, of co-transfected expression constructs for SF1, ERα, hERβ or bERβ at 1x, 2x or 3x concentrations, and 10 nM estradiol (E2). The basal vector pGL3-B served as negative reporter control; the empty vector p0GH served as control for the expression vectors. **(B)** Dose-response relationship for added E2 on the full-length promoter-reporter construct bI3-FL co-transfected with an expression construct for ERα. **(C)** Dose-response relationship for added E2 on the full-length promoter-reporter construct bI3-FL co-transfected with an expression construct for bovine ERβ. **(D)** Dose-response relationship for added E2 on the full-length promoter-reporter construct bI3-FL co-transfected with an expression construct for human ERβ. ^*^Indicates significant difference from control (*p* < 0.05).

In a first set of experiments, bI3-FL was transfected into bovine primary TC, together with either SF1, ERα, or ERβ expression constructs in varying amounts (Figure 8A). Whereas, SF1 indicated a significant stimulation independently of estradiol, ERα showed a marked estradiol-dependent response, with the estradiol following a typical dose-response relationship and an EC50 of 1 nM (Figure 8B). Both bovine and human ERβ expression constructs had been used. Interestingly, neither construct indicated any stimulatory effect on the bINSL3 gene promoter (Figure 8A), and additional estradiol in fact appeared to have either an inhibitory effect (bovine ERβ; Figure 8C), which was independent of the dose applied, or no impact (human ERβ; Figure 8D) except at very high concentration (1 μM).

**Figure 9:**
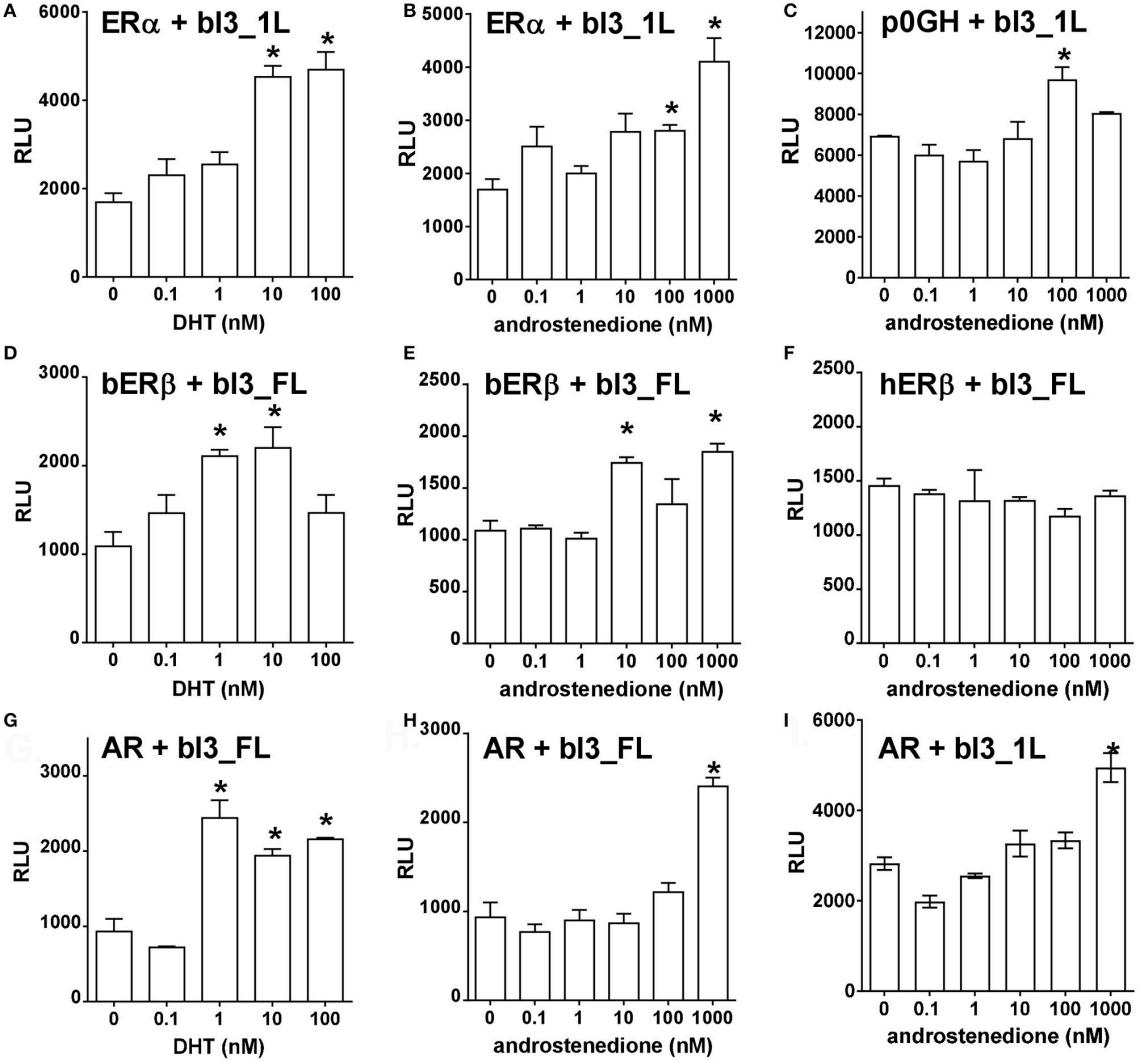
Dose-response effects of different agonists, as indicated, on either bI-FL or bI-1L full-length promoter reporter construct activity (relative light units, RLU) in bovine theca interna cells in the presence of different co-transfected expression vectors for ERα **(A,B)**, bERβ **(D,E)**, hERβ **(F)** or the androgen receptor (AR; **G,H,I**). The vector p0GH served as negative control **(C)**. ^*^Indicates significant difference from vehicle control (*p* < 0.05).

In a second set of experiments, ERα, ERβ (human and bovine), as well as AR were co-transfected with a full-length bINSL3 promoter-reporter construct again into bovine primary TC, and stimulated or not with either androstenedione or DHT (Figure 9). For ERα, both DHT and androstenedione showed a statistically significant stimulation of luciferase activity, though only at higher concentrations (Figures 9A,B). In fact, androstenedione was able to stimulate luciferase activity from the full-length construct even in the absence of added estrogen receptor (Figure 9C), suggesting that this stimulation may be due to endogenous steroid receptors. For bovine ERβ, again both DHT and androstenedione appeared to show a stimulatory effect (Figures 9D,E), which was absent for the human ERβ (Figure 9F). Co-transfection of AR indicated again a significant stimulation by DHT, and also by androstenedione, but for the latter only at the highest concentration (Figures 9G–I). Results were equivalent whether using the constructs bI3_FL or bI3_IL (cf Figures 9H,I).

**Figure 10:**
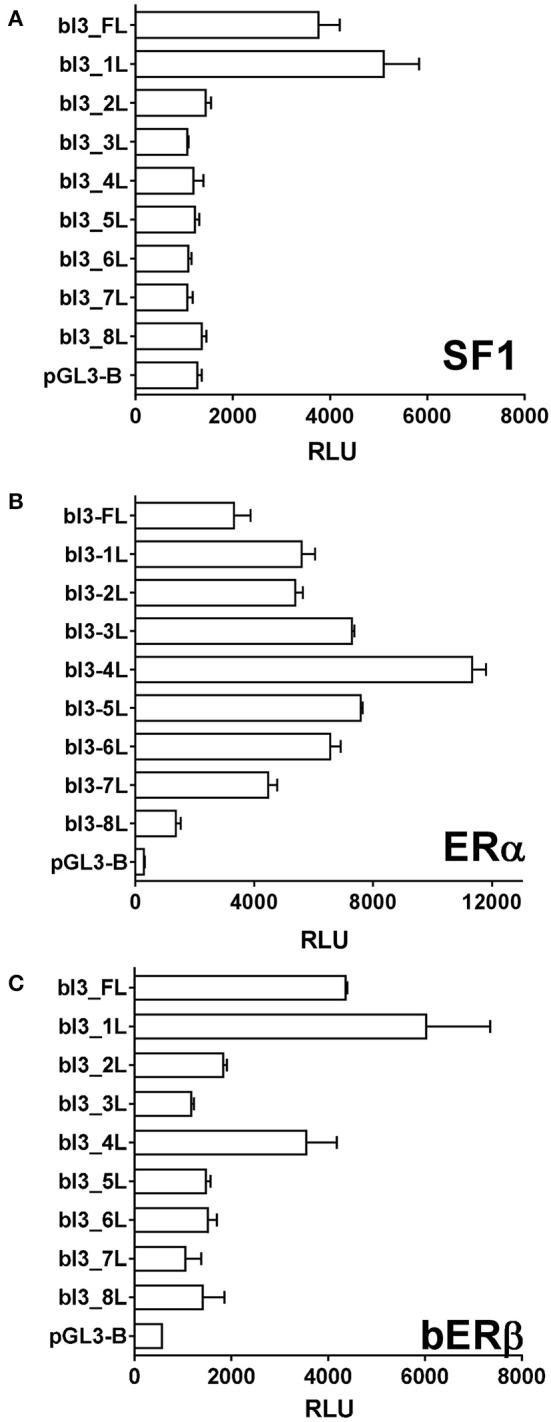
Luciferase activity (relative light units, RLU) generated in bovine theca interna cells transfected with the various deletion-promoter reporter constructs as indicated in Figure 7. **(A)** Co-transfected with the expression vector for SF1. **(B)** Co-transfected with the expression vector for ERα. **(C)** Co-transfected with the expression vector for bovine ERβ.

Finally, deletion promoter-reporter constructs (Figure 7B) were used in conjunction with expression vectors for either SF1, ERα, or ERβ (Figure 10). For SF1, activity was achieved only with the full-length promoter-reporter constructs bI3-FL and bI3-1L (Figure 10A). All shorter constructs were inactive. For E2-activated ERα, in contrast, all deletion constructs except for bI3-8L retained activity (Figure 10B), though notably construct bI3-4L showed maximum activity, implying an inhibitory effect in the more extended promoter constructs. As outlined above (Figure 8C), ERβ differs from ERα by being active on the full-length bINSL3 promoter-reporter constructs even in the apparent absence of exogenous ligand. In the presence of estradiol, the full-length constructs, bI3_FL and bI3_1L, are consistently more active than all deletion constructs (Figure 10C).

## Discussion

*In vivo* INSL3 has been shown to be a valuable biomarker for Leydig cell functional capacity in the male mammal (Ivell et al., 2013), including in the fetus to monitor the impact of environmental endocrine disruption on male fetal development (Anand-Ivell and Ivell, 2014). One of the key features responsible for its increasing application is the observation that in an acute context it appears to be secreted in a constitutive manner and independently of fluctuating LH levels (Ivell and Anand-Ivell, 2009), though this gonadotropin will influence Leydig cell differentiation in the long term. However, relatively little is known about the regulation of INSL3 by steroid hormones. Estrogens have been shown to inhibit INSL3 production by rat and mouse fetal Leydig cells *in vivo* (Emmen et al., 2000; Zhang et al., 2009) presumably by impacting on Leydig cell differentiation. In mouse Leydig cells, estradiol was also shown to be inhibitory on transfected human and mouse INSL3 gene promoter-reporter constructs (Lague and Tremblay, 2009), and appeared to inhibit endogenous INSL3 mRNA levels in these cells, though another study failed to show such an effect (Sadeghian et al., 2005). Similarly, androgens have also been suggested to impact positively on transfected INSL3 promoter-reporter constructs in mouse Leydig cells, whilst having little or no effect on the endogenous mRNA (Lague and Tremblay, 2009). Recently, we have shown that INSL3 is also important for female reproduction, being an essential component in the follicular production and regulation of steroid hormones (Anand-Ivell et al., 2013; Glister et al., 2013). In particular, INSL3 is made exclusively by the theca interna cells (TC) of growing antral follicles and in an autocrine/paracrine manner is responsible for the generation by these cells of androstenedione (Glister et al., 2013), which is the key precursor for most follicular steroid production, including estrogens. In fact, application of siRNA to substantially reduce the expression by bovine TC of either INSL3 or its specific receptor RXFP2 led to almost complete inhibition of androstendione production (Glister et al., 2013). Unlike mature Leydig cells, TC are in a state of continual differentiation across the estrous cycle, responding to the cyclic changes in gonadotropin production by the pituitary and probably also to local hormones from the growing follicle. The present study was undertaken using the bovine model to examine the way in which INSL3 from TC might be regulated by steroids from the local ovarian environment, especially as these might be present in high and varying local concentrations.

The bovine INSL3 gene on chromosome 7, like that from other mammals, is immediately preceded in the genome by the JAK3 gene, whose last exon is only 1,800 bp upstream of the INSL3 transcription start site. As indicated for other species, because of the apparently independent regulation of JAK3, it seems likely that most regulatory DNA elements will be within those 1,800 bp of the immediate upstream promoter region of the INSL3 gene. Inspection of that region, as well as the entire downstream region occupied by the INSL3 coding region and intervening sequence, show there to be no obvious canonical responsive elements recognizing the nuclear receptors for the sex steroid hormones. Instead, as in other species there are three responsive elements for the transcription factor steroidogenic factor 1 (SF1; Figure 7) besides several so-called estrogen-receptor half-sites.

Firstly, we looked at effects of steroid hormones on endogenous INSL3 production by cultured bovine TC, as reflected in peptide secretion. The TC were selected from mid-phase growing antral follicles so that any variation in differentiation status at the beginning of culture could be minimized. These are the cells which are highly steroidogenic, LH-dependent, and indirectly responsible for the high levels of estradiol produced by the granulosa cells of the mid-phase bovine follicle. Unlike for Leydig cells, estradiol had a stimulatory effect on endogenous INSL3 production with an optimum in the low nanomolar range. Importantly, by itself the pure estrogen receptor antagonist, ICI-182,780, was inhibitory on INSL3 production implying the presence in the cell culture of endogenous estrogens (estrogen receptor agonists). Like estradiol, exogenous androstenedione also stimulated INSL3 production, an effect which was similarly inhibited by ICI-182,780 (not shown). Although androstenedione tends to be thought of as a weak androgen, in fact it can act not only at the androgen receptor, but also very effectively at both ERα and ERβ (Miller et al., 2013). In contrast, the androgen receptor antagonist bicalutamide had no significant effect on the androstendione-dependent stimulation of INSL3 production by TC (not shown). In fact, bicalutamide alone appeared to stimulate INSL3 secretion at moderate concentrations, though having no effect at lower and higher concentrations. This would imply that endogenous androgens, acting through the androgen receptor, may in fact be inhibiting INSL3 production. This would also be in line with the results of adding DHT to the cultures, where it appears to have little or no effect. Finally, addition of the progesterone antagonist RU486 led to a marked inhibition of INSL3 secretion, implying the probable involvement also of endogenous progesterone in INSL3 production, generated via the Δ4 pathway. Thus, bovine TC appear to be different from rodent Leydig cells in terms of the way in which they are regulated by sex steroids. This is not surprising given the profile of ambient steroids present in the adult testis. Together with the lack of canonical steroid responsive elements in the INSL3 gene promoter, this suggests that steroid action is less likely to be via classical regulatory mechanisms acting directly on INSL3 gene transcription, but more likely to be indirect by influencing other aspects of cell metabolism or differentiation, or using non-classical signaling pathways (Ivell et al., 2014).

This notion was reinforced also by the experiments using LH or 8Br-cAMP. As shown previously for Leydig cells (Sadeghian et al., 2005), and also for bovine TC (Bathgate et al., 1999; Glister et al., 2013), both substances at low dose had only a modest stimulatory effect, if at all, whereas high doses, equivalent to what might be experienced during the LH surge, were inhibitory. Most significantly, however, any positive effect of low dose LH or 8Br-cAMP was completely negated by the estrogen receptor antagonist ICI-182,780. And contrarily, the stimulatory effect of estradiol on INSL3 secretion could be substantially reduced by the PKA antagonist H89. These findings place the putative interaction between the PKA pathway and the estrogen receptor pathway in the cytoplasm, all of the results together suggesting a complex system much as has been described for breast or prostate cancer cells (Migliaccio et al., 2000, 2005).

In order to explore possible direct effects on INSL3 gene transcription further, we made use in bovine TC of transiently transfected promoter-reporter constructs derived from the bovine INSL3 gene together with expression vectors for several steroid receptors. Here it should be noted that the transfected constructs were likely to be influenced also by both endogenous transcription factors as well as by endogenous steroids, and thus that results need to be interpreted in this context. Generally, most stimulatory activity appeared to reside in the full-length promoter-reporter constructs, where it appears that the region of the most distal SF-1 responsive element at −1,700 bp is playing a key role. Interestingly, responsiveness to activated ERα appears to be contributed by most of the promoter region with maximal effect between −100 and −1,200 bp, a region where previously specific TC nuclear protein binding had been observed (Suhrbier, 2000). Importantly, ERβ appears to behave differently from ERα. Over-expression of the former seems to require the full-length promoter, though curiously its basal activity is inhibited by addition of estradiol. These experiments need to be regarded cautiously since the TC used for these experiments still express endogenous steroid receptors. At higher steroid concentrations both DHT and androstenedione appeared to stimulate the full-length promoter-reporter constructs, when co-transfected with either ERα, ERβ or the androgen receptor (AR). However, it is important to note that both exogenous steroids appear to exert stimulatory effects even when neither added steroid receptors nor cognate responsive elements are present, implying that these transfection experiments may be confounded by endogenous signaling systems. Another important observation from these experiments is that androstenedione is more effective at lower concentration via the transfected estrogen receptors (Figure 9; 10–100 nM) than via the androgen receptor (Figure 9; 1,000 nM), confirming that in this follicular context we should regard this steroid more as an estrogen than an androgen.

Taken together, these results support a model whereby most steroid effects may be occurring within the cytoplasm, impacting on stimulatory pathways which lead either to the specific distal SF1 site, presumably occupied by SF1, or an analog such as nur77, and/or to other transcription factors impacting on the intermediate region of the promoter. There is little direct evidence to support any specific and direct interaction of steroid receptors with the bovine INSL3 promoter region. These results show for mid-cycle bovine TC that estradiol, probably acting through ERα, stimulates INSL3 gene activity, and that androstenedione likely acts in a similar way also through estrogen receptor pathways and not via androgen receptors. Androgens in contrast, at least with endogenous receptor levels, may inhibit INSL3 expression, thus explaining the stimulatory impact of the androgen receptor antagonist bicalutamide. Significantly, low dose LH or dBr-cAMP is convergent with the estrogen pathway since the former pathway can be antagonized by ICI-182,780 and reciprocally estrogen-dependent activation of bINSL3 can be blocked by the PKA inhibitor H89.

The peptide hormone INSL3 is special in that it represents a major non-steroid-related TC product whose expression is thereby independent of conventional steroid metabolism and thus allows some insight into the way local steroids might influence TC function. It should be noted that the INSL3 gene promoter is similar in several ways to the promoter regions of major steroidogenic enzyme genes, such as that for 17α-hydroxylase, which also relies on a prominent SF1-responsive element (Sewer et al., 2008) and is stimulated via a PKA-dependent pathway (Sewer et al., 2008). This study shows that in the context of follicular development and function there is a possibility for estradiol (presumably from granulosa cells), progesterone, and androstenedione (from TC) to positively influence INSL3 expression, and conversely that androgens such as testosterone (also from TC) might counter these effects. Given the role of high levels of estradiol in promoting antral follicle maturation, this suggests a positive feedforward system also involving TC and INSL3 to orchestrate and coordinate antral follicle development. Such a system would give rise to a steadily increasing concentration of circulating estradiol during the follicular phase, which would then be interrupted by the LH surge, when on the one hand cells would luteinize and shift to a Δ4 rather than Δ5 pathway with a high progesterone, low estradiol environment, and INSL3 from TC would be inhibited. The latter was shown here *in vitro* and previously also *in vivo* (Bathgate et al., 1996).

Together with our previous results showing that 17α-hydroxylase expression in bovine theca cells is dependent on activated RXFP2 (Glister et al., 2013), these results emphasize the essential importance of an INSL3-RXFP2 positive feedforward loop, which is further promoted by increasing estrogen (estradiol, androstenedione) concentrations, not only on INSL3 secretion but most importantly on steroidogenesis as a whole via concomitant stimulation of 17α-hydroxylase.

We still understand relatively little about the intracellular mechanisms within TC which integrate and orchestrate the various signaling pathways involved in INSL3 production and action. Their elaboration at the molecular level must await further research.

These results are also relevant for our understanding of the steroidal consequences in polycystic ovarian syndrome (PCOS), where antral follicles appear to be arrested in an early differentiation state, with limited aromatase expression and high levels of secreted steroid precursors (Franks et al., 2000) as well as INSL3 (Anand-Ivell et al., 2013). The apparently higher expression of both 17α-hydroxylase and the LH receptor within individual theca cells from affected ovaries (Comim et al., 2013), the consideration that the syndrome may be due to altered ovarian steroidogenesis (Franks et al., 1999), together with the association of certain INSL3 polymorphisms with PCOS incidence (Shaikh et al., 2016), suggest that targeting the INSL3/RXFP2 system in the ovary could offer a novel therapeutic intervention.

## Author contributions

YD carried out most of the experimental work, and assisted in conception, writing and interpretation of the results. RI was responsible for conception of the project, bioinformatic analysis, interpretation of results and writing of the manuscript. RA-I had overall responsibility for the project, contributed to conception, experimental design, individual experiments, results interpretation, and writing of the manuscript.

### Conflict of interest statement

The authors declare that the research was conducted in the absence of any commercial or financial relationships that could be construed as a potential conflict of interest.
